# Supplementary material for: Detecting changes in generation and serial intervals under varying pathogen biology, contact patterns and outbreak response
Source: PLoS Comput Biol. 2024 Mar 22;20(3):e1011967. doi: 10.1371/journal.pcbi.1011967 (PMC10990235; doi:10.1371/journal.pcbi.1011967)
Supplement: S1 Text — (DOCX) [file pcbi.1011967.s001.docx]

# **S1 Text**

**Detecting changes in generation and serial intervals under varying pathogen biology, contact patterns and outbreak response**

Rachael Pung^1,2^*, Timothy W. Russell^2^, Adam J. Kucharski^2^

^1^Ministry of Health, Singapore

^2^Centre for the Mathematical Modelling of Infectious Diseases, London School of Hygiene and Tropical Medicine

*Corresponding author: [rachael.pung@lshtm.ac.uk](mailto:rachael.pung@lshtm.ac.uk)


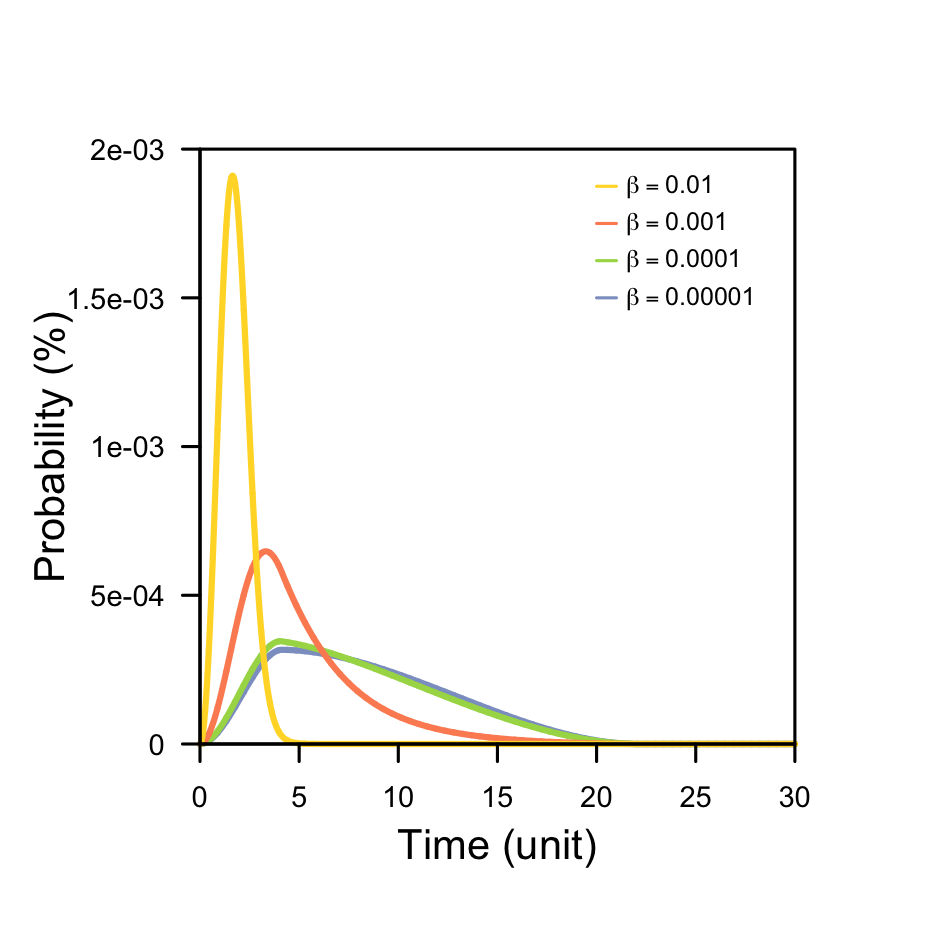


**Figure A** Probability of infection at different timestep. Peak infectiousness profile varied based on $\beta$ scale factor which influences the time of peak probability of infection. At low values of $\beta$, the distribution of probabilities is approximately similar over time.


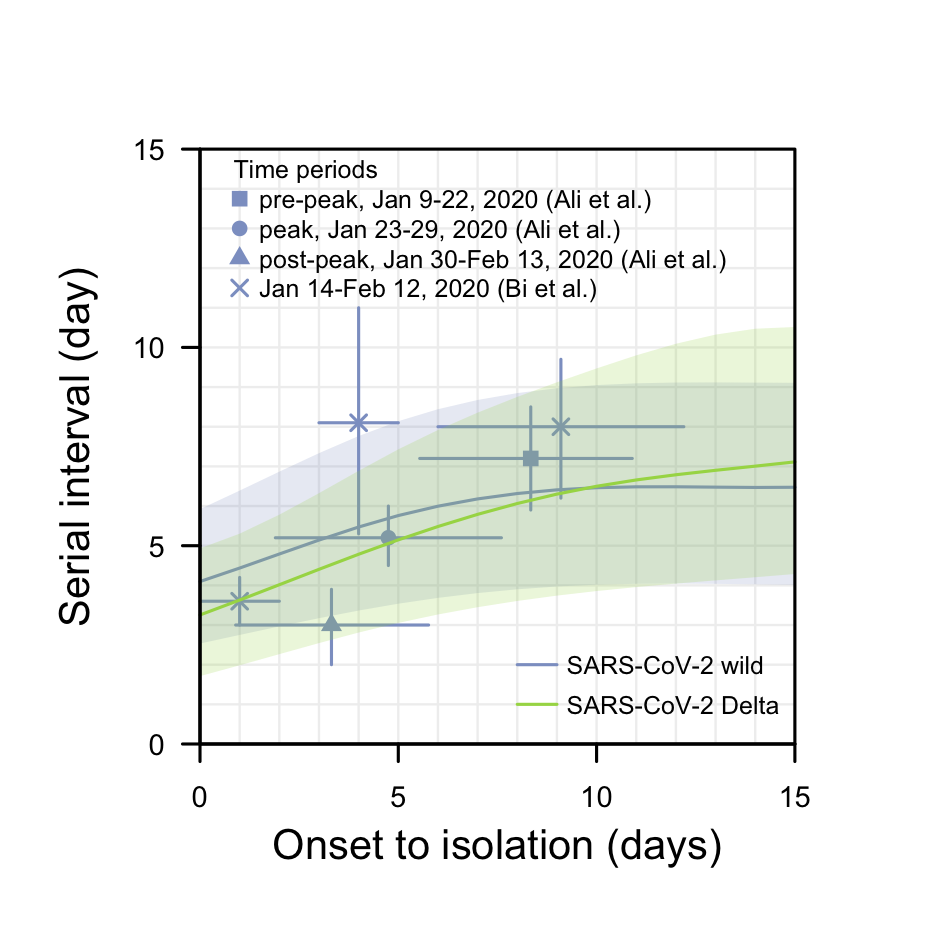


**Figure B**. Modelled serial interval for varying delay in case onset-to- isolation in SARS-CoV-2 wild type and Delta variant with median (lines) and interquartile range (shaded regions). We assumed higher peak infectiousness for the Delta variant and the attack rate for the Delta was twice that of the wild type in the absence of isolation. Observed serial intervals from published studies [12,14] as shown in points (mean) with lines (95% CI).


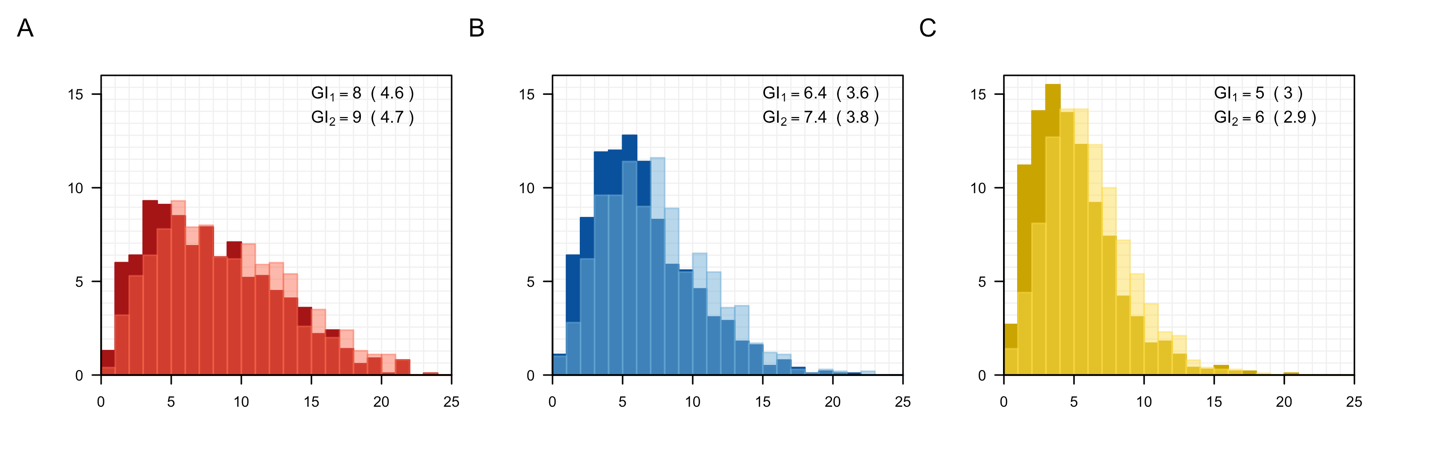


**Figure C** Histogram and mean (sd) of generation intervals in reference (dark, GI_1_) and alternative (light, GI_2_) pathogen under (A) no isolation, (B) case isolation on average 8 days post symptoms onset, (C) case isolation on average 4 days post symptoms onset.


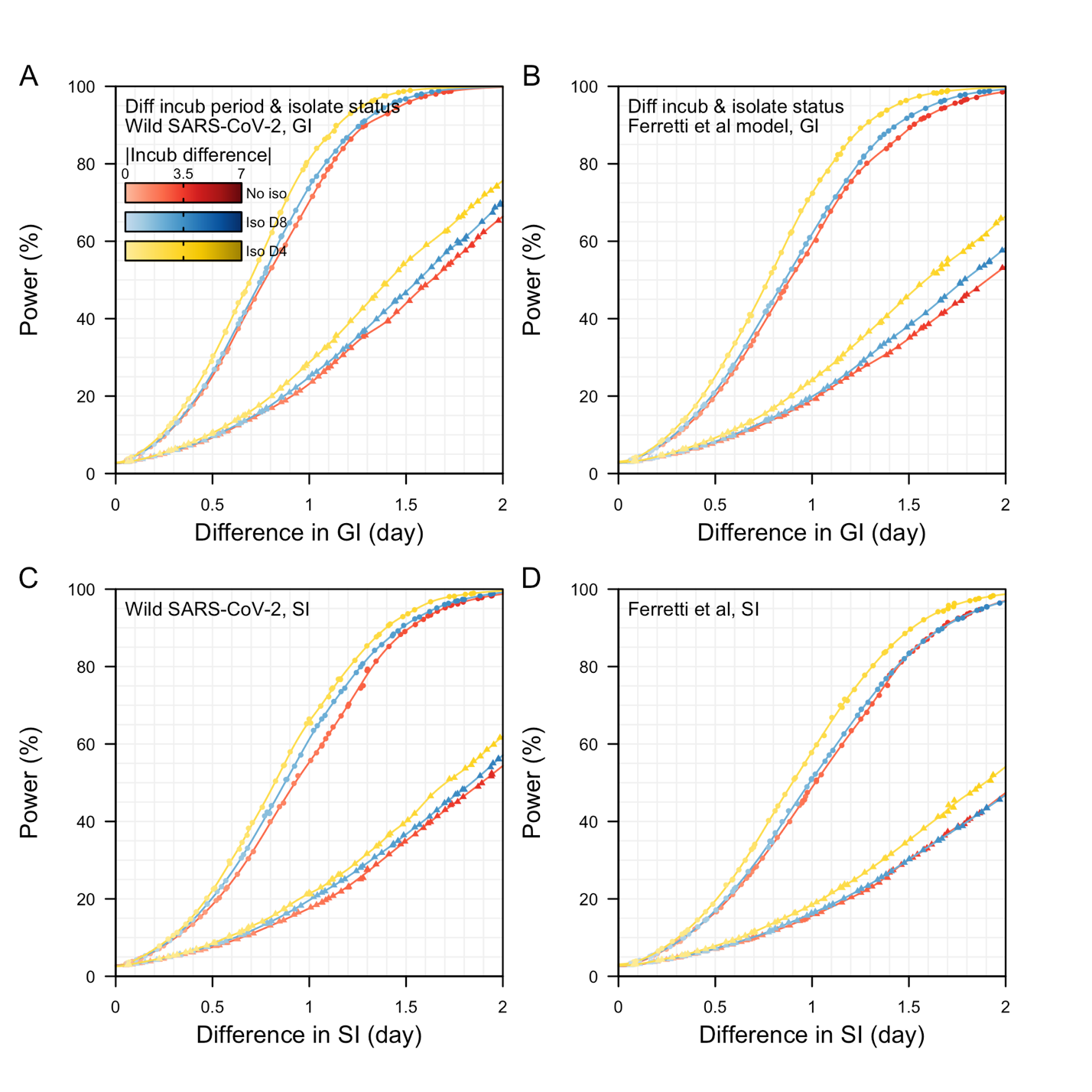


**Figure D** Power to detect differences in generation (GI) and serial (SI) intervals between reference and emerging pathogen. (A,C) Different incubation period between reference and alternative pathogen under same symptoms onset-to-isolation status of either no isolation, mean symptoms onset-to-isolation is 8 days, or 4 days using spline model; (B,D) similar to (A,C) but using skew logistic model by Ferretti et al [9].


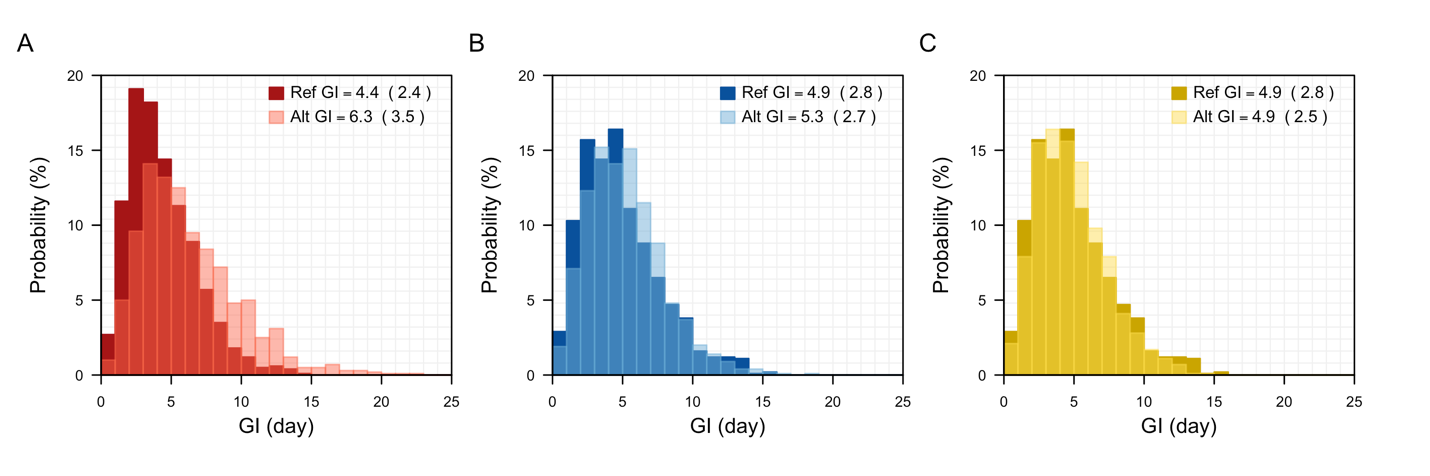


**Figure E** Generation intervals, GI, without adjusting for bias introduced by different epidemic dynamics for refence pathogen with a one-day shorter incubation period and longer shedding duration as compared to alternative pathogen. (A) exponential growth in reference pathogen but exponential decline in alternative pathogen, (B) constant growth in both reference and alternative pathogen, (C) constant growth in reference pathogen but exponential growth in alternative pathogen.


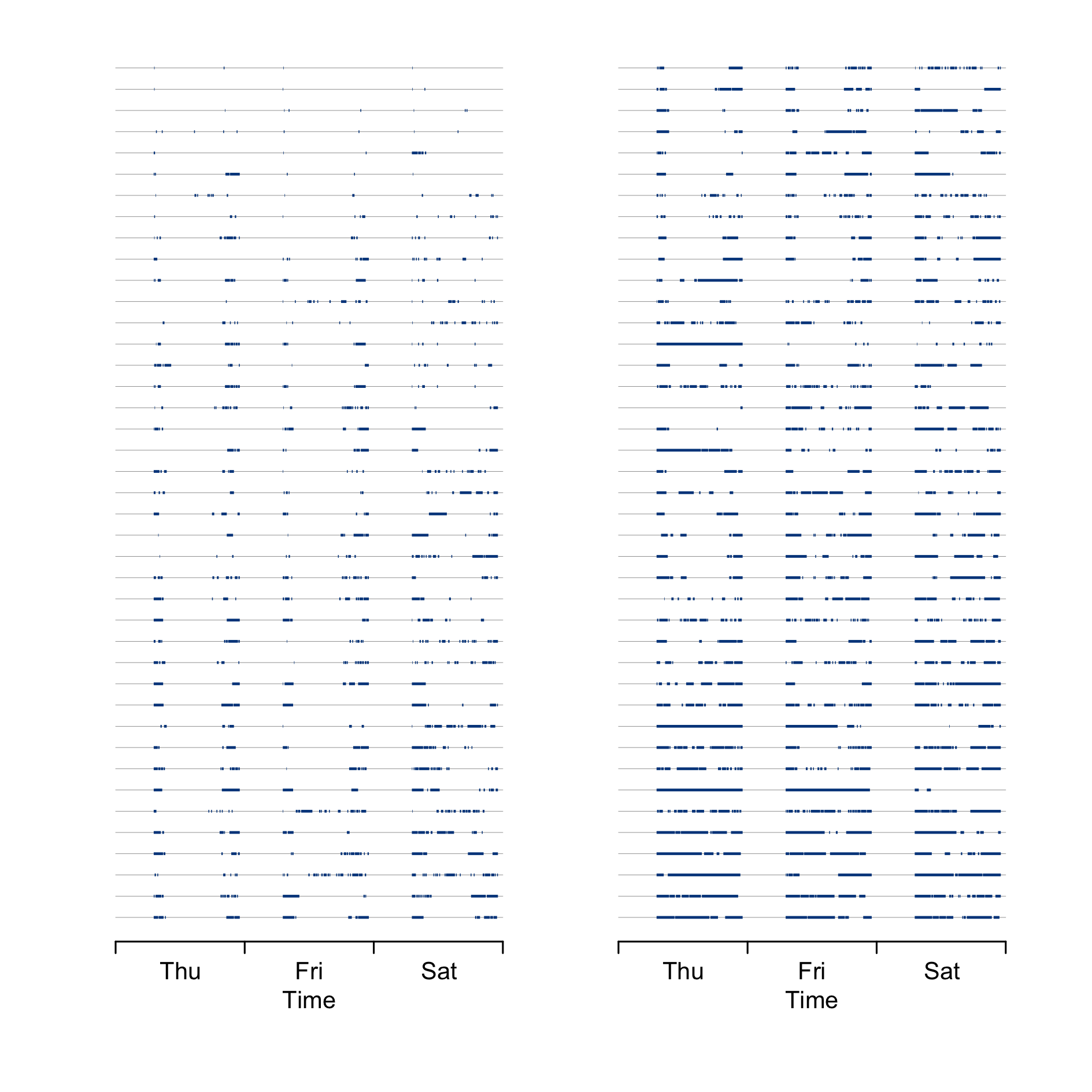


**Figure F** Contact sequence at different times of day. Each line represents a pair of individuals with recorded contact in a five-minute time interval (blue bars). Data was not collected from 2300-0700 hours.

**Table A** Differences in mean incubation period of reference (Delta-like) and alternative (wild type-like) pathogen for a one day difference in mean generation interval when sample size is 100. Power to detect this difference in incubation period as shown in brackets. Peak infectiousness of Delta-like reference pathogen was scaled by $\beta$with values of 0.0005, 0.002, 0.006. The corresponding probability of infection was 20%, 50% and 80% when the mean incubation period was 4 days and peak infectiousness coincided with symptoms onset. Peak infectiousness of wild type SARS-CoV-2-like alternative pathogen is scaled by $\beta$of 0.0005. Duration of infectiousness after the peak infectiousness is 8 days shorter in the reference pathogen.

| Onset-to-isolation | | 4 days post onset on average | No isolation |
| --- | --- | --- | --- |
| Probability of infection of Delta-like reference pathogen | 20% | 1.9 (71%) | 5.0 (39%) |
|  | 50% | 1.3 (73%) | 2.9 (45%) |
|  | 80% | 0.2 (85%) | 0.0 (64%) |

**Table B** Differences in mean incubation period of reference (Delta-like) and alternative (wild type-like) pathogen for a one day difference in mean generation interval when sample size is 100. Power to detect this difference in incubation period as shown in brackets. Peak infectiousness of Delta-like reference pathogen was scaled by $\beta$with values of 0.0005, 0.002, 0.006. The corresponding probability of infection was 20%, 50% and 80% when the mean incubation period was 4 days and peak infectiousness occurs between 0-2 days prior to symptoms onset. Peak infectiousness of wild type SARS-CoV-2-like alternative pathogen is scaled by $\beta$of 0.0005. Duration of infectiousness after the peak infectiousness is 8 days shorter in the reference pathogen. Negative difference indicates that the incubation period of reference pathogen is larger than alternative pathogen.

| Onset-to-isolation | | 4 days post onset on average | No isolation |
| --- | --- | --- | --- |
| Probability of infection of Delta-like reference pathogen | 20% | 1.5 (72%) | 4.9 (35%) |
|  | 50% | 1.3 (74%) | 2.6 (38%) |
|  | 80% | 0.4 (81%) | -1.1 (56%) |

**Text A**

The relationship of the generation and serial interval can be expressed as follow [15,16]:

$$S_{ij}= P_{ij}+I_{j}$$

$$G_{ij}= P_{ij}+I_{i}$$

$$G_{ij}= S_{ij}+I_{i}-I_{j}$$

where $S_{ij}$ is the serial interval between infector $i$ and infectee $j$, $P_{ij}$ is the onset-to-transmission, $I_{i}$ and $I_{j}$ are the incubation period of infector $i$ and infectee $j$ and $G_{ij}$ is the generation interval.

The variance of the generation interval can be expressed as:

$$Var\left( S \right)=Cov\left( S,S \right)$$

$$=Cov\left( P_{ij}+I_{j},P_{ij}+I_{j} \right)$$

$$=Cov\left( P_{ij},P_{ij} \right)+2Cov\left( P_{ij},I_{j} \right)+Cov\left( I_{j},I_{j} \right)$$

Assuming that the onset-to-transmission in the infector is independent with the incubation period of the infectee (i.e. $Cov\left( P_{ij},I_{j} \right)=0$), thus,

$$Var\left( S \right)=Var\left( P_{ij} \right)+Var\left( I_{j} \right)$$

The variance of the generation interval can be expressed as:

$$Var\left( G \right)=Cov\left( G,G \right)$$

$$=Cov\left( P_{ij}+I_{i},P_{ij}+I_{i} \right)$$

$$=Cov\left( P_{ij},P_{ij} \right)+2Cov\left( P_{ij},I_{i} \right)+Cov\left( I_{i},I_{i} \right)$$

$$=Var\left( P_{ij} \right)+2Cov\left( P_{ij},I_{i} \right)+Var\left( I_{i} \right)$$

Assuming that the incubation period distribution of the infector and infectee is independent and identically distributed, thus

$$Var\left( I_{i} \right)=Var\left( I_{j} \right)$$

and

$$Var\left( G \right)=Var\left( S \right)+2Cov\left( P_{ij},I_{ij} \right)$$
