# Supplementary material for: Detecting changes in generation and serial intervals under varying pathogen biology, contact patterns and outbreak response
Source: PLoS Comput Biol. 2024 Mar 22;20(3):e1011967. doi: 10.1371/journal.pcbi.1011967 (PMC10990235; doi:10.1371/journal.pcbi.1011967)
Supplement: Attachment — Submitted filename: PCOMPBIOL-D-23-01002 Reviewers Comment.pdf [file pcbi.1011967.s002.pdf]

Ms. Ref. No.: PCOMPBIOL-D-23-01002

Title: Detecting changes in generation and serial intervals under varying pathogen biology, contact patterns and outbreak response

Comments:

I have read this paper in a detailed manner. I wish to state that your work on Detecting changes in generation and serial intervals under varying pathogen biology, contact patterns and outbreak response is commendable. But a lot needs to be done. Therefore, author needs to do overall corrections of grammar and spelling if this manuscript will be accepted. Also, consider the following corrections listed below:

1. This manuscript is not structure well into Chapters/ Sections the paper supposes should be structured.
2. You did not consider the existence, uniqueness and properties of the model formulated for this work.
3. Numerical stimulated is absent from this work and so on.

There are many things to be done on this manuscript but if it will be accepted, I would direct the authors to study and include the following papers for more clarification:

- Analysis and Dynamics of Fractional order Mathematical Model of COVID-19 in Nigeria using Atangana-Baleanue operator, *Computers*, *Materials & Continua*, (2020).
- A new mathematical model of COVID-19 using real data from Pakistan. Results Phys 2021; 24:104098. doi:10.1016/j.rinp.2021.104098
- Forecasting of COVID-19 Pandemic in Nigeria Using Real Statistical Data. Communication in Mathematical Biology and Neurosciences. 2021 (2021). doi:10.28919/cmbn/5144.
- Mathematical model of COVID-19 in Nigeria with optimal control, *Results in Physics*, **28**(2021), 104598. doi: 10.1016/j.rinp.2021.104598
- A fractional-order mathematical model for malaria and COVID-19 co-infection dynamics, *Healthcare Analytics*, **4**(2023), 100210. doi: 10.1016/j.health.2023.100210
