# Supplementary material for: Detecting changes in generation and serial intervals under varying pathogen biology, contact patterns and outbreak response
Source: PLoS Comput Biol. 2024 Mar 22;20(3):e1011967. doi: 10.1371/journal.pcbi.1011967 (PMC10990235; doi:10.1371/journal.pcbi.1011967)
Supplement: Attachment — Submitted filename: plos_reviewer_reply_v2.docx [file pcbi.1011967.s003.docx]

Dear Editorial team and reviewers,

Thank you very much for coordinating the review and for the comments. The following is a point-by-point reply to each comments.

Best regards,

Rachael

----------------------------------------------

**Comments to the Authors:
Please note here if the review is uploaded as an attachment.**

Reviewer #1: Comments:
I have read this paper in a detailed manner. I wish to state that your work on Detecting changes in generation and serial intervals under varying pathogen biology, contact patterns and outbreak response is commendable. But a lot needs to be done. Therefore, author needs to do overall corrections of grammar and spelling if this manuscript will be accepted.

Thank you for the comments and we have corrected the grammatical and spelling errors accordingly.

Also, consider the following corrections listed below:
1.This manuscript is not structure well into Chapters/ Sections the paper supposes should be structured.
Thank you for highlighting this and we have structured the revised manuscript into the Plos Computational Biology format for the main body (i.e. Introduction, Results, Discussion, Materials and Methods).

2.You did not consider the existence, uniqueness and properties of the model formulated for this work.

Although it is common to study existence and uniqueness in theoretical analysis of equilibrium solutions to dynamical systems, particularly those composed of ordinary differential equations, this is not the topic of our analysis. The main model used in this study simulates stochastic transmission between a pair of individuals only and does not consider further transmission beyond the secondary case. This is unlike an ODE model which explores the effect of different parameters on equilibrium solutions, particularly thresholds for non-zero final epidemic sizes (i.e. R0>1). Because we are interested in the statistical power to correctly estimate generation and serial intervals, we instead explore the interaction between pathogen biology, contact pattern and early isolation policies, and how they collectively affect the generation and serial intervals in independent transmission pairs.

3.Numerical stimulated is absent from this work and so on.
As mentioned in the previous comment, the objective of the study was to simulate independent transmission pairs and to estimate the change in generation or serial intervals given changes in different pathogen biology, contact patterns or isolation policies. So in this sense, numerical simulations are included. However, full transmission models are beyond the scope of our research question so we do not show full simulations of epidemics.

There are many things to be done on this manuscript but if it will be accepted, I would direct the authors to study and include the following papers for more clarification:
• Analysis and Dynamics of Fractional order Mathematical Model of COVID-19 in Nigeria using Atangana-Baleanue operator, Computers, Materials & Continua, (2020).

Study on COVID-19 transmission dynamics by measuring the reproduction number in Nigeria. The authors also identified the force of infection and progression rate from exposed to infected compartments as key model parameters influencing the extent of the outbreak.

•A new mathematical model of COVID-19 using real data from Pakistan. Results Phys 2021; 24:104098. doi:10.1016/j.rinp.2021.104098

Similar study as above but using Pakistan’s data.

•Forecasting of COVID-19 Pandemic in Nigeria Using Real Statistical Data. Communication in Mathematical Biology and Neurosciences. 2021 (2021). doi:10.28919/cmbn/5144.

Use of time series regression models to predict the short-term COVID-19 incidence in Nigeria and compare with the observed incidence.

•Mathematical model of COVID-19 in Nigeria with optimal control, Results in Physics, 28(2021), 104598. doi: 10.1016/j.rinp.2021.104598

Using a compartmental model to project outbreak trajectory under different combinations of outbreak control strategies such as (i) use of face mask, hand sanitizer and social distancing, (ii) screening, testing and case isolation, (iii) measure to minimise recurrence or reinfection in previously infected persons.

•A fractional-order mathematical model for malaria and COVID-19 co-infection dynamics, Healthcare Analytics, 4(2023), 100210. doi: 10.1016/j.health.2023.100210

Simulation study on the co-infection of COVID-19 and malaria in Nigeria highlighting that preventive measures for one disease have knock-on effect on preventing another disease

We have reviewed the above papers and they do not seem relevant to the topic of our study. We have provided brief summaries above in gray, but can reconsider if the reviewer provides details of why these papers are pertinent (and more relevant than any other references). Moreover, we noticed that the same authors appear on all the papers, which seems unusual – we would like to check that there is no conflict of interest with the reviewer proposing this set of papers to be cited.

Reviewer #2: In this article, the authors explore how generation and serial intervals depend on various factors, such as changes in infection characteristics and isolation strategies. They further calculate statistical power for estimating the differences in generation and serial intervals across a wide range of scenarios. While this study is a useful contribution, I have some concerns.

Even though the calculation of statistical power is a major component of this study, the methods for power calculation is not explained anywhere in the main text or in supplementary materials. The calculation of statistical power depends on the statistical model as well as the assumed significance level. Moreover, I'm more concerned about the reported powers for the generation intervals. In reality, generation intervals are rarely observed. Instead, they are estimated from the observed serial intervals. Propagating uncertainties in the estimation of generation intervals from serial intervals are likely to decrease the power. If the authors are calculating the power to detect differences in the mean generation interval, assuming that generation intervals are known exactly, they are likely overestimating the power.

Thank you for the comments. First, we have now added a “Statistical test” subsection within the Material and Methods section. Second, we have also expanded our exploration of the relationship between generation interval and serial interval. In the initial version, we estimated the power to detect changes in the generation interval assuming perfect knowledge of the transmission pairs and the power to detect changes in the corresponding observed serial interval. However, in reality, generation intervals are rarely observed and would need to be inferred using the observed serial intervals. Thus, in the revised version, we used two inference approaches to infer the mean and variance of the generation interval (i.e. inferred generation interval) from the observed serial intervals.

We assumed two extreme scenarios when inferring the generation intervals from the observed serial intervals: the infectiousness of an infector is (i) dependent on the time of symptoms onset of the infector or (ii) dependent on the time of infection of the infector and independent of symptoms [15].

For (i), the timing of transmission concentrates around the time of the symptoms onset. The incubation period of the infector and the time from onset-to-transmission is independent. The sum of both quantities gives the generation interval and if we assume both infector and infectee have the same incubation period, then the variance of the inferred generation interval is the same as the variance of the serial interval.

For (ii), the infectiousness profile is independent of the onset of symptoms in the infector. Thus, the timing of transmission is not correlated with the timing of symptoms onset. The generation interval here is the sum of the delay from infection to infectiousness and the time from infectiousness to transmission. The corresponding variance of the inferred generation interval is lower than the observed serial interval.

The two extreme assumptions would result in same mean but different variance in the inferred generation interval distribution and forms the upper and lower limits of the power to changes in the inferred generation interval if serial interval is used as a proxy [15]. We have amended the Material and Methods sections; extracted below for easy reference.

## *Statistical test*

*We used a two sample Welch’s t-test to estimate the power to detect a difference in means of the interval distributions of the reference and alternative pathogen. The test was two-sided with a significance level of 5%. An equal number of intervals were sampled from each distribution (25 or 100 samples out of 1,000 simulated pairs) and distributions are of unequal variances.*

*In reality, generation intervals are rarely observed and would need to be inferred using the observed serial intervals [15]. We studied two inference approaches: that the infectiousness of an infector is (i) dependent on the time of symptoms onset of the infector or (ii) dependent on the time of infection of the infector [15]. In each approaches, we would infer the mean and variance of the generation interval (i.e. inferred generation interval) based on the assumptions elaborated in [15] and Supplementary Information. Using this mean and variance, we will perform the Welch’s t-test to obtain the estimated power to detect a difference in the means of the inferred generation interval distribution of the reference and the alternative pathogen.*

*In both approaches, we assumed that the incubation period distribution of the infector and the infectee are independent and identically distribution. As such, the inferred generation interval and observed serial interval have the same mean. The relationship between the variance of the inferred generation interval can be expressed broadly as follows [15] with further elaboration in the supplementary info:*

$$Var(G) = Var(S) + 2Cov(P_{ij},I_{i})$$

*(5)*

*where* $G$ *is the generation interval,* $S$ *is the serial interval,* $P_{ij}$ *is the onset-to-transmission between infector* $i$ *and infectee* $j$*,* $I_{i}$ *is the incubation period of infector* $i$*. In the first inference approach, we assumed the incubation period of the infector and the time from onset-to-transmission are independent (i.e.* $Cov(P_{ij},I_{i})=0$*). Hence, the variance of the inferred generation interval is reduced to:*

$$Var(G) = Var(S)$$

*(6)*

*In this approach, the inferred generation interval has the same mean and variance as the observed serial interval, and is the same as the serial interval.*

*In the second assumption, the infectiousness profile is independent of the timing of symptoms (i.e. timing of transmission is not correlated with the timing of symptoms,* $Cov(G,I_{i})=0$*) and the variance of the inferred generation interval can be expressed as:*

$$Var(G) = Var(S) + 2Cov(P_{ij},I_{i})$$

$= Var(S) + 2Cov(G-I_{i},I_{i})$

$= Var(S) + 2[Cov(G-I_{i})-Cov(I_{i},I_{i})]$

$= Var(S) - 2Var(I)$

$\leq$ $Var(S)$

*(7)*

*Thus, the generation interval can be more broadly defined as the sum of the time from infection to infectiousness in the infector and the time from infectiousness to infection. The variance of the inferred generation interval is thus smaller than the observed serial interval.*

*Both are extreme approaches and formed the basis of inferring the upper and lower limits of the variance of the inferred generation intervals (i.e. lower and upper limits of the power to detect a difference in the inferred generation intervals) [15].*

Under the results section (which comes before the methods section), we have also expanded our initial analysis on the power to detect changes in the theoretical generation interval and observed serial intervals, and included the power to detect changes in the inferred generation interval distributions based on the above inference approaches. Below is a summary of the amendments:

*As generation intervals are rarely observed, serial intervals are often used as a proxy for the delay between generations of infection. In general, the mean of the inferred generation interval is similar to the mean of the observed serial interval when the infector and infectee have the same incubation period distribution [15,16]. The variance of the inferred generation interval is dependent on the variance of the serial intervals and on the covariance of the onset-to-transmission and incubation period of the infector. To understand how the changes in the variance affect the power to detect differences in the inferred generation intervals, we therefore explored two extreme scenarios based on [15].*

*At one extreme, we assumed that the infectiousness of an infector is dependent on the time of symptoms onset only. Under this assumption, the incubation period of the infector and the time from onset-to-transmission are independent and their sum equates to the generation interval. For the same incubation period distribution in the infector and infectee, this assumption implies that the generation interval is the same as the serial interval (sum of time from onset-to-transmission in infector and incubation period of infectee). Thus, the corresponding power to detect the difference in the inferred generation and observed serial intervals are equivalent (Figure 2b).*

*At the other extreme, we assumed that the infectiousness is dependent on the time since infection of the infector. As such, the time of transmission is not correlated with the time of the time of symptom onset in the infector and the variance of the derived generation interval is lower than the observed serial interval. The two assumptions serve as the upper and lower limits to the variance of the inferred generation interval (i.e. lower and upper limits of the power). Under the second assumption, the power to detect a one-day difference in the generation interval for a sample size of 100 was 46% with no isolation, 75% with onset-to-isolation of 8 days and 100% for onset-to-isolation of 4 days (Figure 2c). These power values are higher than under the assumption of the lower limit for GI (Figure 2b), but in practice this could also increase the chance of a false positive (i.e. concluding a difference in GI when there is not none).*

To recap, we simulated a scenario where the incubation period of the alternative pathogen was 1.35 days higher than the reference pathogen, a delay from onset-to-isolation of 4 days and similar contact patterns for pairs of individuals infected with the reference and alternative pathogen. The theoretical power to detect a one-day difference in the generation interval was 66% for an onset-to-isolation of 4 days (Fig A below, black cross). The power to detect a difference based on observed serial intervals was 54% and forms the lower limit of the power to detect changes in the inferred generation interval (Fig B below, black cross). The upper limit of this power estimated based on the second assumption of the variance of the inferred generation interval was 100% (Fig C below, black cross)

X

X

X

[15] Lehtinen S, Ashcroft P, Bonhoeffer, S. 2021 On the relationship between serial interval, infectiousness profile and generation time. *J. R. Soc. Interface.*182020075620200756 [http://doi.org/10.1098/rsif.2020.0756](https://doi.org/10.1098/rsif.2020.0756)

Moreover, it seems like the effects of epidemic dynamics have been largely overlooked in this study. Typically, when a new variant invades a population, the previous variant in typically in a declining state. As the authors explain in the introduction, differences in epidemic growth rates can further translate to differences in the observed serial intervals. It would be interesting and useful to understand how these biases affect power and the observed differences.

In response to this comment, we have added a new section in on “Epidemic dynamics” in the Materials and Methods and “Different epidemic growth dynamics of variants” in the Results and added further discussion on our outcomes. There are three parts to our analysis - (i) outcomes to the generation intervals when we do not adjust for different epidemic dynamics in the reference and alternative pathogen, (ii) the corresponding power to detect changes without adjustments, and (iii) corresponding power after adjustments.

*Figure 4 Generation intervals, GI, under varying outbreak dynamics. (A) Differences in the generation interval between the reference and alternative pathogen without adjusting for exponential growth or decline outbreak dynamics. Exponential growth of 0.2/day (red), constant outbreak (blue) and exponential decline of 0.2/day (yellow) in alternative pathogen; (B) corresponding power to detect the biased differences in the generation intervals; (C) power to detect differences in the generation intervals after correctly adjusting for exponential outbreak dynamics.*

We developed the first part of the analysis based on the method in Park et al, 2021 [11]. In Fig 4A, we illustrated the difference in the mean generation interval under varying exponential growth/decline rate of the reference pathogen (x-axis -0.3-0.3/day) and the alternative pathogen (red: exponential growth of 0.2/day, blue: constant outbreak, yellow: exponential decline of 0.2/day). The blue dot is the expected (true) difference in the generation interval between both pathogens in the absence of exponential outbreak dynamics. The red dot shows a likely scenario of a new emerging variant undergoing exponential growth and an existing variant experiencing exponential decline. The yellow dot shows another scenario of one variant undergoing exponential growth while another experiencing stable dynamics.

We elaborated in the results sections as follow:

*The power to detect a difference in the generation intervals is dependent on the extent of overlap in the generation interval distribution of the reference and alternative pathogen. The extent of this overlap is in turn affected by the differences in the biological characteristics of each pathogen. However, this overlap also depends on the prevailing outbreak dynamics. Without adjusting for exponential growth and decline dynamics in the reference and alternative pathogen respectively, the extent of overlap in the generation interval distributions of the reference and the alternative pathogen is lesser as compared to the scenario where both pathogens are at constant incidence (Supplementary Figure 6a and 6b). This accentuates the differences in the mean generation interval, thereby increasing the power to detect this difference and conclude that there exist non-zero difference between the generation intervals of two pathogens (i.e. lower Type II error) (Figure 4b, red square). Furthermore, unadjusted outbreak dynamics can also increase the chance of concluding a difference in the generation intervals when there is none after adjustment (i.e. higher Type I error). However, when we correctly adjust for the exponential outbreak dynamics, we recovered a similar mean difference in the generation intervals of the reference and alternative pathogen across different combinations of epidemic dynamics, and a similar power to detect this difference (Figure 4c).*

*Overall, the sample size of a study should be designed based on the desired power to detect a difference after adjusting for the observed epidemic dynamics. Without adjusting for epidemic dynamics, there is a possibility of accentuating the differences in the generation intervals for a reference pathogen undergoing exponential growth and an alternative pathogen experiencing exponential decline. Consequently, even with a small sample size there is a large power to detect this biased difference (Figure 4b). However, after adjusting for the epidemic dynamics, the power to detect the inherent difference in the generation intervals would be reduced (Figure 4c). Thus, it is important to account for epidemic dynamics when planning for the appropriate number of samples for collection under the prevailing or likely outbreak dynamics.*

Minor comment
- L52 "When variant prevalence grows rapidly within a population, it may be the result of increased transmissibility" It's unclear that increased transmissibility should necessarily correlate with shorter generation intervals. One could imagine a pathogen with higher R0 but identical infectiousness profile (and therefore generation interval distribution). It would be useful to distinguish generation intervals in real epidemics vs those representing infectiousness profiles

We wish to clarify that the intention of this sentence was to highlight that the observed growth of cases could be attributed to a mix of factors influencing transmissibility or the timescales of infection (i.e. generation intervals). We did not intend to imply anything about correlation of transmissibility and generation intervals within this sentence. The example provided by Review #2 is valid, but in this instance not directly related to the point we were trying to make.

The full sentence in the introduction is reproduced below for reference:

“When variant prevalence grows rapidly within a population, it may be the result of increased transmissibility, or a shorter delay from one infection to the next, or both.”

Furthermore, we would like to elaborate on the comparison of generation intervals in real epidemics vs studies on infectiousness profiles. In another study [1], we have compared the serial intervals (as generation intervals are hard to measure) of the wild-type and the Delta variant in a COVID-19 epidemic in Singapore. From the study, we did not observe a significant difference of more than one-day in the serial interval. However, the infectiousness profile was not monitored in these transmission pairs. Thus, it was difficult to determine if there was a change in the peak viral load, viral shedding duration and hence infectiousness profile, and whether these changes cancelled each other out or interacted with other external factors. As such, this motivated the current study to explore different infectiousness profile and its impact on generation and serial intervals.

[1] https://www.thelancet.com/journals/lancet/article/PIIS0140-6736(21)01697-4/fulltext

- L61 "Observed serial intervals may be shorter during the exponential phase of an outbreak because transmission events involving
infectees with longer incubation periods have yet to be observed" This is incorrect. Serial intervals may be longer during the growth phase because infectors are more likely to have shorter incubation periods (when we consider a group of infectors who developed symptoms at the same time)

Thank you for this comment.

Our statement as written is technically correct, because there are scenarios where the observed intervals may be shorter. But as the reviewer notes, there may also be certain combinations of circumstances where a longer period may be measured.

During an epidemic growth phase, infectees with shorter incubation periods are more likely to be observed earlier. Hence, the observed serial intervals will be shortened when considering the onset times of the infectees (i.e. serial intervals **completed** by a given time). This is because the serial interval is the sum of the onset to transmission delay for the infector and the incubation period for the infectee.

In order to interpret these intervals correctly, we agree with the reviewer that one should measure the serial intervals of infectors of the same onset time (i.e. serial intervals **started** by a given time). The corresponding serial interval would generally be longer than the aforementioned observed serial intervals. This was explored in a study by Park et al. and was the same citation [11] provided in this sentence in L61.

[11] <https://www.pnas.org/doi/abs/10.1073/pnas.2011548118>

We have made the following edits for clarity:

Observed serial intervals based on the onset times of the infectee ~~may~~ would be shorter during the exponential phase of an outbreak because transmission events involving infectees with longer incubation periods have yet to be observed [11].

- Overall, the infectiousness profile needs to be explained more clearly

We have elaborate this in the next comment.

- L133: "We then fitted a cubic Hermite spline at the respective time points and constrained the slope of the spline to be zero at each point to simulate the infectiousness profile over the course of the infection" This sentence needs some unpacking

We have edited this line as follows:

For each individual, we simulated the start and end time of the infectiousness period, with time of peak infectiousness for respective diseases relative to the incubation period (Table 1). The relative infectiousness ranged from 0 to 1 — normalised relative to the peak infectiousness. We then fitted a cubic Hermite spline through the start, peak and end points of the infectiousness period. ~~at the respective time points and~~ We constrained the slope of the spline to be zero at each of the three points (i.e. first derivative is zero) to simulate the infectiousness profile over the course of the infection. Furthermore, we scaled the splines of respective disease such that the probability of infection matches observed data (Table 1).

To illustrate, the slopes of the infectiousness profile below is zero (equivalent to a horizontal tangent) at the start, peak and end of the infectiousness profile.

- Equation 1: it's not obvious that the exp(Lambda(t-1)) term is needed or what it's trying to capture. Equation 1 would approximate lambda in the absence of the exp(Lambda(t-1)) term

Thank you for the comment. To recap equation is as follow:

$$p_{inf}(t) =e^{-\Lambda(t-1)}\left( 1-e^{-\lambda(t)} \right)$$

(1)

$$\approx\lambda(t)$$

In this rearranged version of eq 1, $e^{-\Lambda(t-1)}$ on the RHS is the probability of not being infected since the start of the infector’s infectiousness to timestep t-1. $\left( 1-e^{-\lambda(t)} \right)$ is the probability of being infected in timestep t. This allows us to calculate the probability of infection occurring between two individuals over respective time steps spanning the duration of infectiousness (e.g. 10 days after symptoms onset in wild-type SARS-CoV-2, Table 1). The stochastic model then samples the time step of infection based on these probabilities. We have added the following clarification with the “Simulating transmission” subsection:

The first coefficient on the RHS of Equation 1 is the probability of surviving infection up to time step $t-1$ and the second coefficient is the probability of being infected at time step $t$. The stochastic model then samples the time of infection in each pair of individuals based on $p_{inf}$.

- Simulating transmission: how many days were epidemics simulated for? Until the epidemic dies out naturally?

We wish to clarify that the simulated transmission occurred in a pairs of infector and infectee only. We did not propagate the transmission beyond the infectee in the main analysis. As such, the simulation between a pair of individuals stops once a transmission occurred successfully. Under the “Simulating transmission” subsection we have added the following:

Once the simulated transmission occurred between a pair of individuals, there was not further propagation of the infection. We simulated 1,000 transmission pairs for each combination of pathogen and epidemiological characteristics.

- "Corresponding power to detect these differences under varying human contact patterns and outbreak response" How was the power calculated? Is there an underlying statistical model?

Thank you for highlighting this and as mentioned in the earlier replies, we have added a subsection on “Statistical test” within the Material and Methods section.

**Have the authors made all data and (if applicable) computational code underlying the findings in their manuscript fully available?**
The [PLOS Data policy](https://journals.plos.org/ploscompbiol/s/materials-and-software-sharing) requires authors to make all data and code underlying the findings described in their manuscript fully available without restriction, with rare exception (please refer to the Data Availability Statement in the manuscript PDF file). The data and code should be provided as part of the manuscript or its supporting information, or deposited to a public repository. For example, in addition to summary statistics, the data points behind means, medians and variance measures should be available. If there are restrictions on publicly sharing data or code —e.g. participant privacy or use of data from a third party—those must be specified.

Reviewer #1: **No:** Data is not available on this manuscript

Reviewer #2: **No:** I couldn't find access to the code used for simulating epidemic or calculating the power.

We have now made the codes available at https://github.com/rachaelpung/generation_intervals
