# Supplementary material for: Detecting changes in generation and serial intervals under varying pathogen biology, contact patterns and outbreak response
Source: PLoS Comput Biol. 2024 Mar 22;20(3):e1011967. doi: 10.1371/journal.pcbi.1011967 (PMC10990235; doi:10.1371/journal.pcbi.1011967)
Supplement: Attachment — Submitted filename: plos_reviewer_reply_part2_v1.docx [file pcbi.1011967.s004.docx]

Dear Editorial team and reviewers,

Thank you for the review and additional comments. The following is reply to reviewer #2’s comment on power calculations.

Best regards,

Rachael

----------------------------------------------

Reviewer #2: The authors have addressed all my previous concerns. My only remaining concern is about the power calculations for the inferred distributions. Specifically, the authors state that they estimate the mean and variance of the generation interval distribution from the serial interval distribution and calculate power based on a t test. However, it doesn't seem like the authors account for the uncertainties associated with inference, which would overestimate the power. I don't necessarily suggest that the authors perform a new set of analyses but this seems like an important limitation of the current approach.

Thank you for this comment and we have provided further clarification and discussion under the limitation section.

Secondly, we did not account for variations in the start and end times of the infectiousness profile, and instead fixed these parameters based on average observed durations of viral growth or decline. Furthermore, the scale factor for the peak infectiousness were not modelled based on a distribution. Accounting for these variations are not likely affect the mean difference in the generation and serial intervals or the parameters (e.g. mean incubation period) that result in this difference but will lead to reduced power to detect these differences. We estimated the power to detect a difference in the generation intervals with a Welch t-test using the estimated serial intervals for the reference and alternative pathogen. We considered two bounding assumptions about the population-level relationship between the variance of the generation intervals and serial intervals to obtain a plausible range of power values. However, in reality, the inference method for obtaining generation intervals distributions could introduce additional uncertainty. If we were to instead try and infer this relationship from individual transmission pairs, it would be important to account for the resulting parameter uncertainty to avoid underestimating the variance of the distributions and hence the power.

Figure Files:

Converted and uploaded

Data Requirements:

All data stored in GitHub repository <https://github.com/rachaelpung/generation_intervals/tree/main/output>

Reproducibility:

All methods documented within the main manuscript and codes are uploaded in GitHub repository. No laboratory processes involved in this study.

References:

Reviewed and not aware of any retracted papers being cited
